# Supplementary material for: Salicylic Acid-Producing Endophytic Bacteria Increase Nicotine Accumulation and Resistance against Wildfire Disease in Tobacco Plants
Source: Microorganisms. 2019 Dec 22;8(1):31. doi: 10.3390/microorganisms8010031 (PMC7022923; doi:10.3390/microorganisms8010031)
Supplement: Supplementary file 1 [file microorganisms-08-00031-s001.pdf]

Supplementary Materials

Table S1. Quantification of salicylic acid production by endophytic bacteria isolated from various plant species based on HPLC method.

**Plant species**	**Isolates**	**Isolates Identified as**	**Tissues**	**SA production (µg/mL)**
Taxus brevifolia	EB-2	*Micrococcus luteus*	Fruit	NM*
	EB-7	*Enhydrobacter aerosaccus*	Leaves	NM
	EB-17	*Brachybacterium rhamnosum*	Leaves	NM
Salix babylonica	EB-42	*Pseudomonas syringae pv. phaseolicola*	Leaves	NM
	EB-43	*Xanthomonas campestris pv. campestris*	Leaves	NM
	EB-44	*Pseudomonas tremae*	Leaves	57.05
	EB-45	*Xanthomonas campestris pv. campestris*	Leaves	NM
	EB-46	*Pantoea vagans*	Leaves	NM
	EB-47	*Curtobacterium herbarum*	Leaves	46.22
	Eb-48	*Curtobacterium plantarum*	Leaves	NM
	EB-49	*Agrobacterium tumefaciens*	Leaves	NM
	EB-50	*Xanthomonas gardneri*	Leaves	44.24
	EB-51	*Bacillus altitudinis*	Leaves	NM
	Eb-53	*Pseudomonas rhizosphaerae*	Leaves	NM
	EB-55	*Curtobacterium citreum*	Leaves	NM
	EB-56	*Agrobacterium tumefaciens*	Leaves	NM
	EB-57	*Curtobacterium flaccumfaciens pv. Flaccumfaciens*	Leaves	NM
	EB-58	*Curtobacterium plantarum*	Leaves	NM
	EB-59	*Pseudomonas psychrotolerans*	Leaves	NM
	EB-60	*Curtobacterium flaccumfaciens pv. flaccumfaciens*	Leaves	NM
	EB-62	*Agrobacterium larrymoorei*	Leaves	NM
	EB-63	*Enhydrobacter aerosaccus*	Leaves	NM
	EB-65	*Brevundimonas vesicularis*	Leaves	NM
Metasequoia glyptostroboides	EB-83	*Pseudomonas syringae* pv. *phaseolicola*	Cones	NM
	EB-84	*Micrococcus luteus*	Cones	NM
Ginkgo biloba	EB-87	*Staphylococcus hominis* subsp. *novobiosepticus*	Leaves	NM
	EB-124	*Bacillus subtilis* subsp. *subtilis*	Fruits	NM

*NM-Not measurable amount of salicylic acid produced by endophytic bacteria, because minimum peak height was less than 0.1mV.
